# Sex differences in ischemic heart disease and evidence gathering related to exposure risk, prevention, and treatment of per- and poly-fluoroalkyl substances

**DOI:** 10.3389/fpubh.2025.1596125

**Published:** 2025-06-20

**Authors:** Hejun Tian, Xiaofei Huang

**Affiliations:** Department of Cardiology, Jiangxi Provincial People’s Hospital, The First Affiliated Hospital of Nanchang Medical College, Nanchang, China

**Keywords:** ischemic heart disease, per- and poly-fluoroalkyl substances, molecular docking, molecular dynamics simulations, legacy toxicity, restricted cubic spline

## Abstract

**Objective:**

To investigate the sex differences in environmental exposure to per- and poly-fluoroalkyl substances (PFAS) in ischemic heart disease (IHD) and to identify potential targets for future prevention and treatment of PFAS-associated IHD.

**Methods:**

The Global Health Data Exchange database was used to explore the sex differences in IHD mortality and morbidity. The National Health and Nutrition Examination Survey (NHANES) database was used to identify sex differences in response to environmental exposure to PFAS, including survival probability and dose–response. The Comparative Toxicogenomics Database and Gene Expression Omnibus databases were used to search for critical signaling pathways involved in IHD pathogenesis and potential targets for the prevention and treatment of PFAS-associated IHD. The binding stability of these complexes was evaluated by molecular docking and molecular dynamics simulations.

**Results:**

Globally, the mortality, morbidity, years of life lost, and years lived with disability are higher for men than women. Among 42,742 participants from NHANES, including IHD and control groups as well as PFAS-affected IHD subjects, men had significantly lower survival rates than women. Four PFAS exposures, including perfluorooctane sulfonamide, perfluorooctane sulfonic acid (PFOS), perfluorooctanoic acid (PFOA), and 2-(N-methyl-PFOSA) acetate, significantly worsened the survival of patients with IHD and interacted with 105 human genes associated with cardiovascular diseases. Combining differentially expressed genes from the pluripotent stem cell-derived cardiomyocyte dataset, five promising genes-CASP3, PDK4, GDF15, RPL17, and CTNNB1-were identified as having high binding stability to PFAS.

**Conclusion:**

Men with IHD have significantly worse survival rates than women, yet women are more susceptible to PFOA and PFOS toxicity. This study also identifies several PFAS receptor genes that affect key pathways in IHD pathogenesis, which are promising potential targets for future prevention and treatment of PFAS-associated IHD.

## Graphical abstract

Graphical abstract of evidence survey for this study. 
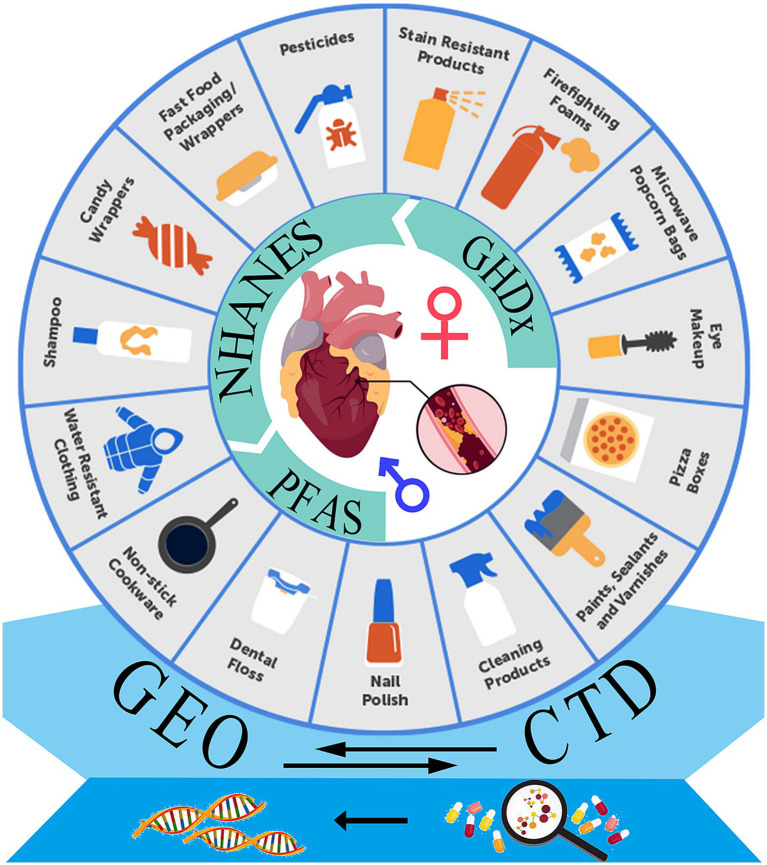

## 1 Introduction

Per- and poly-fluoroalkyl substances (PFAS) are widely used in industrial products and are environmental pollutants associated with the risk of various diseases, such as cardiovascular disease, type 2 diabetes, and thyroid cancer (1–5). Due to their hydrophilic functional groups and hydrophobic alkyl side chains, the high stability and persistence of this class of chemicals lead to their accumulation over time, resulting in legacy PFAS toxicity in animals and humans (6, 7). Therefore, the elimination of PFAS relies heavily on non-metabolic pathways, such as bile acids, urine, and feces (8–10), which are inefficient in scavenging toxic chemicals from the human body and are one of the reasons for the accumulation of PFAS toxicity.

The significant toxicity of PFAS on cardiovascular disease has been reported in hypertension (3, 11), coronary heart disease (12), and dyslipidemia (13). However, these findings are controversial (12, 14). Ischemic heart disease (IHD) is a common cardiovascular disease that leads to hospitalization and death, including coronary heart disease, angina, heart attack, etc. (15, 16). It is widely believed that the deposition of cholesterol and fat in coronary arteries is the main cause of IHD. Its important risk factors include hypertension, hyperlipidemia, smoking, diabetes, obesity, lack of exercise and genetic factors. However, there is still a gap in the relationship between environmental factors (such as PFAS pollutants) and IHD, and the effect of legacy PFAS on the pathogenesis of IHD is unclear. Moreover, the sex differences in toxicity and dose responses to PFAS in patients with IHD have not been studied. Therefore, it is of great significance to further understand the impact of PFAS on IHD and to identify potential targets for the prevention and treatment of PFAS-related diseases in the future.

## 2 Materials and methods

### 2.1 Data collections

As shown in the graphical abstract, the epidemiological data on mortality, morbidity, years of life lost (YLLs), and years lived with disability (YLDs) for global IHD from 1980 to 2021 were downloaded from the Global Health Data Exchange (GHDx). Data on PFAS exposure in participants with or without IHD were collected from the National Health and Nutrition Examination Survey (NHANES) from 1999 to 2018. The effects of exposure to PFAS on gene expression associated with human cardiovascular diseases, including myocardial ischemia, myocardial infarction, coronary artery disease, and angina, were collected from the Comparative Toxicogenomics Database (CTDbase) (17). The toxicity effects of exposure to PFAS on human-induced pluripotent stem cell-derived cardiomyocytes (PSCC) were extracted from GSE262419 (18) of the Gene Expression Omnibus (GEO) database.

### 2.2 Definitions of participants and exposures

All participants, who were 18 years of age or older, were asked the following questions: (a) Have you ever been told you had coronary heart disease? (b) Have you ever been told you had angina/angina pectoris? (c) Have you ever been told you had a heart attack? Any responses marked as “refused” or “forgotten” were considered missing data and removed from the data analysis. Organic fluorochemicals, including per- and poly-fluoroalkyl substances, were detected in accordance with laboratory quality assurance and monitoring rules.

### 2.3 Sex differences in PFAS exposure risk

Kaplan–Meier survival analysis was used to compare sex differences among all participants, as well as IHD patients and controls. Univariate and multivariate Cox proportional hazard models were used to identify the important PFAS associated with survival risk in IHD patients. Quantile and trend comparisons were used to explore the stratification differences of PFAS on survival risk between female and male patients. Restricted cubic spline (RCS) models were used to compare the dose-risk relationships of significant PFAS between sexes. Based on the minimum Akaike information criterion of all RCS models, the optimal knot was selected within an iterative range of 3 ~ 7 knots.

### 2.4 Functional annotation of PFAS-affected genes

The functional enrichment analysis of PFAS-affected human genes, which were downloaded from CTDbase, was performed based on the comprehensive MSigDB 3.0 (19) databases, including the biological processes of gene ontology, the Kyoto Encyclopedia of Genes and Genomes (KEGG), and the Human Phenotype Ontology (HPO). The network of interactions between all affected genes was mapped based on canonical pathways from the KEGG MEDICUS pathway database (20).

### 2.5 Analysis of differentially expressed genes

To determine the effect of PFAS on IHD, we utilized the PSCC *in vitro* experimental dataset GSE262419 (18) to search for differentially expressed genes (DEGs). In the published experiment, we extracted only datasets of cell lines treated with 10uM PFAS and 0uM medium. The relative fold change of gene expression was calculated using the R ‘limma’ package (version 3.58.1) (21).

### 2.6 Molecular docking

Using AutoDock Vina (version 1.1.2) (22), molecular docking was performed to evaluate the effects of important PFAS on target genes through receptor-ligand binding affinity. In each receptor-ligand docking process, the protein structure of the target gene was downloaded from the RCSB Protein Data Bank (PDB),^1^ and its binding position was detected by Fpocket (version 3.0) based on Voronoi tessellation and alpha spheres (23). For each screened binding pocket, the drug score had to be greater than 0.1, and only the 10 optimal docking postures were retained.

### 2.7 Molecular dynamics simulation

The molecular dynamics simulation trajectories of the complexes were generated using GROMACS (2020.3) (24). The PDB structure was prepared using the PDBFixer (version 1.9.0) function at pH 7. A simple point charge was applied to the reaction system in a cubic box (*d* = 1 nm) with an external force field GROMOS96 54A7 (25). The steepest descent minimization algorithm was adopted to minimize the energy of the reaction system for 10,000 steps, and the minimization process was completed when the maximum force was < 10.0 kJ/mol. Finally, 100 ns simulations were performed at 300 K and 1 atm.

### 2.8 Statistical analysis

Statistical analyses such as Kaplan–Meier survival analysis, the establishment of univariate and multivariate Cox proportional hazards models, RCS models, functional enrichment analysis, and DEG analysis, were performed on the R platform (V 4.3.2)^2^ by packages survival (V 3.7.0), rms (V 6.3.0), clusterProfiler (V 4.10.1) and limma (V 3.58.1). Molecular docking and dynamics simulations were performed on Ubuntu 22.04, and the results were visualized using Python (V 3.9.9) packages ‘pymol’ (V 2.5.7) and ‘py3Dmol’ (V 2.0.4).

## 3 Results

### 3.1 Sex differences in IHD epidemiology

**Figure 1:**
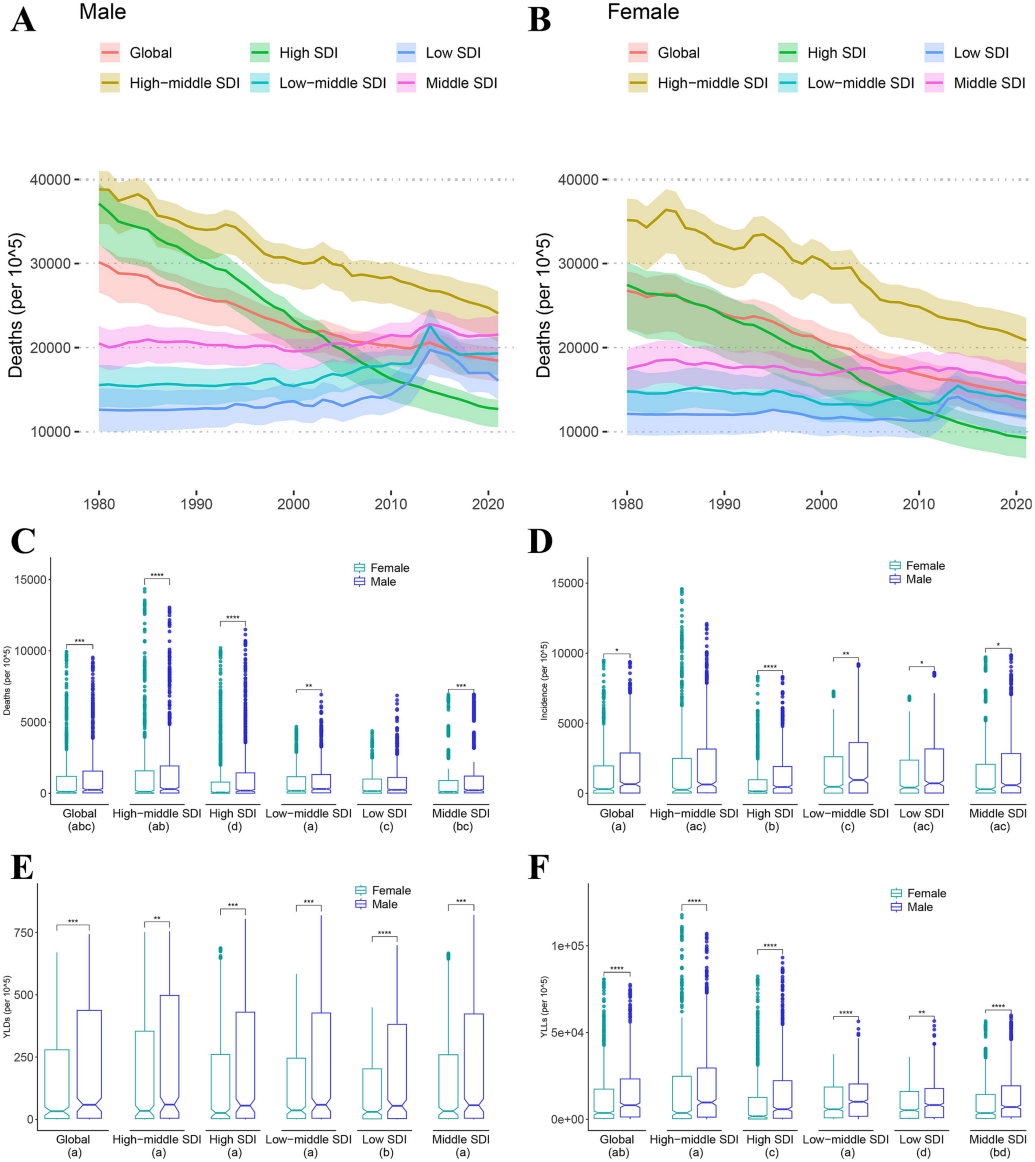
Sex differences of mortality, morbidity, years of life lost and years lived with disability. **(A,B)** Death trends of male **(A)** and female **(B)** from 1999 to 2021 by different Socio-demographic Index (SDI). **(C–F)** Wilcox-test for sex differences in mortality **(C)**, morbidity **(D)**, years lived with disability (YLDs) **(E)** and years of life lost (YLLs) **(F)**. Different letters in each group below indicate a significant difference, and vice versa (* < 0.05, ** < 0.01, *** < 0.001, **** < 0.0001).

As shown in Figure 1, mortality rates for both males (Figure 1A) and females (Figure 1B) are declining globally and in the high SDI and middle-high SDI regions. However, in other regions, including middle SDI, middle-low SDI, and low SDI, the number of deaths did not decrease significantly and even showed a sudden increase around 2015, especially among male populations, reaching more than 20,000 deaths per 10^5.

In the comparison test for sex differences, death rates (Figure 1C) were significantly higher in the male population than in females at all SDI levels except low SDI. The incidence rates (Figure 1D) were significantly higher in the male population than in females at all SDI levels except high-middle SDI. In the comparison of years of life with disability (YLDs, Figure 1E) and years of life lost (YLLs, Figure 1F), the person-year mean of the male population was significantly higher than that of the female population at all SDI levels. Additionally, we found that the YLLs of low SDI is significantly lower than other SDL levels. Similarly, regions with low SDI had significantly lower YLDs than regions with other SDI levels.

### 3.2 Sex differences in risk of exposure to PFAS

**Figure 2:**
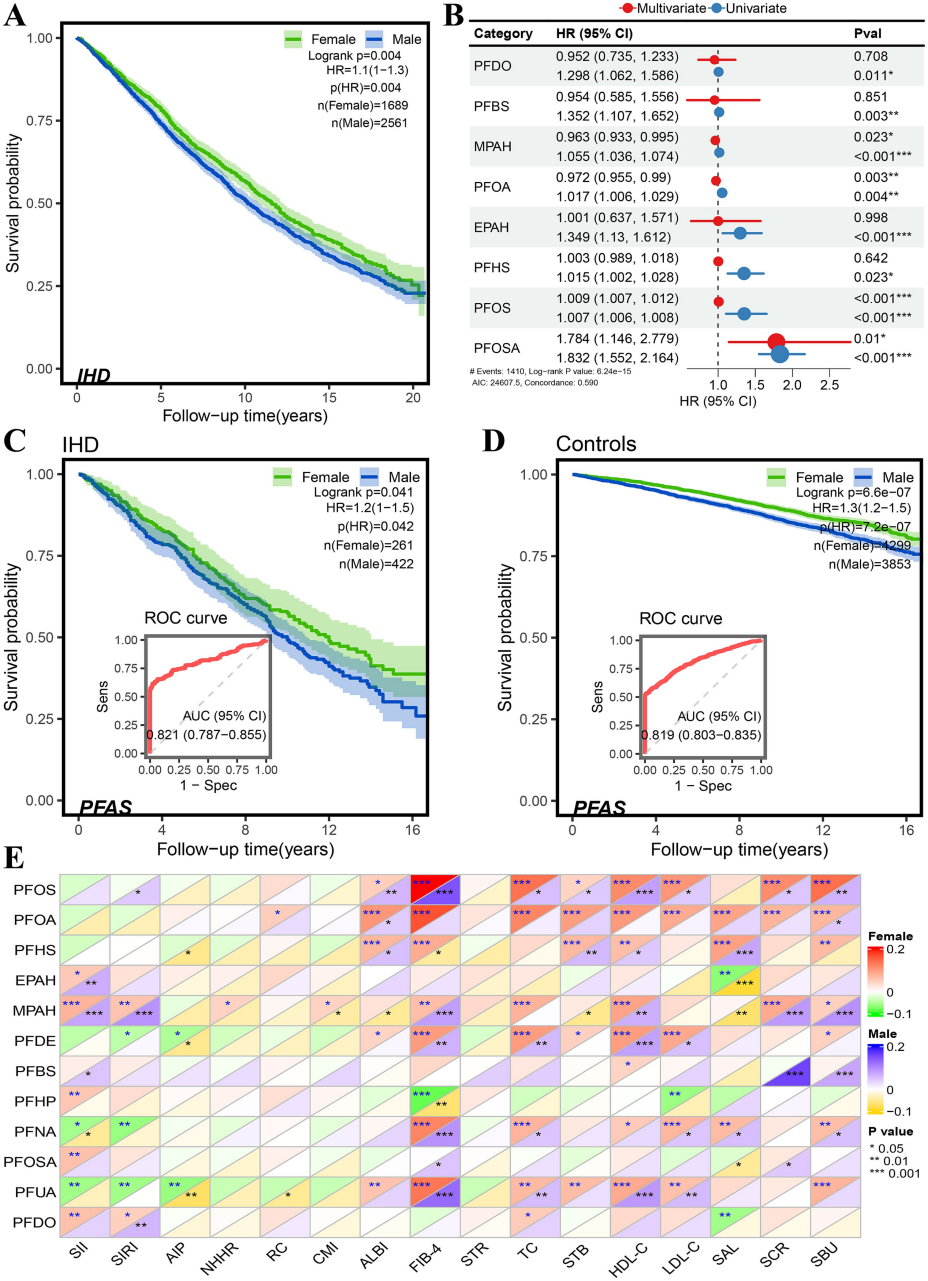
Sex differences in risk associated with PFAS exposure in NHANES database. **(A)** Sex difference in survival probability of IHD patients. **(B)** Forest plot of univariate and multivariate Cox proportional hazard models of PFAS exposure. **(C,D)** Sex difference in survival probability of PFAS exposure among participants, and the performance of PFAS in distinguishing the survival status in IHD patients **(C)** and controls **(D)**. **(E)** Pearson correlation of PFAS with serum and its derived biomarkers (* < 0.05, ** < 0.01, *** < 0.001).

In a total of 10 cohorts of datasets, 3,247 of 42,742 participants (Table 1) were defined as patients with IHD, and the risk of death was significantly higher in men than in women (Figure 2A), HR = 1.1 (95% CI: 1.0–1.3, *p* = 0.004). Of all eligible participants, 8,851 had documented exposure to PFAS, of which 684 had IHD (Table 1). Both univariate and multivariate Cox proportional risk analyses showed that exposure to perfluorooctane sulfonamide (PFOSA), perfluorooctane sulfonic acid (PFOS), perfluorooctanoic acid (PFOA), and 2-(N-methyl-PFOSA) acetate (MPAH) significantly worsened the survival of IHD patients (Figure 2B, Supplementary Figure S1). Details of the PFAS substances was provided in Supplementary Table S1.

**Table 1:** Basic information of participants.

Category	All participants				Test	Participants exposure to PFAS				Test
	*N*	% / Mean (SD)	*N*	% / Mean (SD)		*N*	% / Mean (SD)	*N*	% / Mean (SD)	
IHD	No		Yes			No		Yes		
Age	39,495	48 (18)	3,247	67 (13)	*F* = 3610.034, *p* < 0.001	8,167	48 (18)	684	68 (13)	*F* = 793.554, *p* < 0.001
Gender	39,495		3,247		*X*^2^ = 232.947, *p* < 0.001	8,167		684		*X*^2^ = 53.402, *p* < 0.001
Female	20,844	53%	1,261	39%		4,310	53%	261	38%	
Male	18,651	47%	1986	61%		3,857	47%	423	62%	
Race	39,495		3,247		*X*^2^ = 319.092, *p* < 0.001	8,167		684		*X*^2^ = 65.72, *p* < 0.001
Mexican American	6,518	17%	356	11%		1,398	17%	76	11%	
Non-Hispanic Black	8,664	22%	564	17%		1820	22%	105	15%	
Non-Hispanic White	16,477	42%	1870	58%		3,736	46%	422	62%	
Other Hispanic	3,542	9%	236	7%		628	8%	42	6%	
Other Race	4,294	11%	221	7%		585	7%	39	6%	
Citizenship	39,401		3,247		*X*^2^ = 200.64, *p* < 0.001	8,146		684		*X*^2^ = 55.568, *p* < 0.001
Not US	5,919	15%	193	6%		1,235	15%	32	5%	
US	33,482	85%	3,054	94%		6,911	85%	652	95%	
Marital	39,468		3,246		*X*^2^ = 667.609, *p* < 0.001	8,159		684		*X*^2^ = 145.354, *p* < 0.001
Married or cohabiting	23,622	60%	1818	56%		4,862	60%	385	56%	
Never married	7,499	19%	195	6%		1,550	19%	37	5%	
Widowed, divorced or separated	8,347	21%	1,233	38%		1747	21%	262	38%	
Pregnancy	10,054		146		*X*^2^ = 4.925, *p* = 0.026	2,207		35		*X*^2^ = 1.183, *p* = 0.277
No	9,143	91%	141	97%		1991	90%	34	97%	
Yes	911	9%	5	3%		216	10%	1	3%	
Education	39,444		3,241		*X*^2^ = 140.028, *p* < 0.001	8,149		684		*X*^2^ = 69.346, *p* < 0.001
High school and below	18,951	48%	1908	59%		4,088	50%	457	67%	
More than high school	20,493	52%	1,333	41%		4,061	50%	227	33%	
Total persons in family	39,495		3,247		*X*^2^ = 103.761, *p* < 0.001	8,167		684		*X*^2^ = 27.44, *p* < 0.001
<=5	35,040	89%	3,069	95%		7,236	89%	651	95%	
>5	4,455	11%	178	5%		931	11%	33	5%	
Hypertension	12,832		2,369		*X*^2^ = 177.634, *p* < 0.001	2,592		488		*X*^2^ = 53.956, *p* < 0.001
No	1861	15%	106	4%		397	15%	14	3%	
Yes	10,971	85%	2,263	96%		2,195	85%	474	97%	
Diabetes	39,469		3,246		*X*^2^ = 1471.399, *p* < 0.001	8,160		683		*X*^2^ = 269.035, *p* < 0.001
Borderline	818	2%	110	3%		142	2%	32	5%	
No	34,337	87%	2038	63%		7,191	88%	449	66%	
Yes	4,314	11%	1,098	34%		827	10%	202	30%	
Smoking	39,468		3,246		*X*^2^ = 414.208, *p* < 0.001	8,162		684		*X*^2^ = 90.812, *p* < 0.001
No	22,259	56%	1,230	38%		4,521	55%	249	36%	
Yes	17,209	44%	2016	62%		3,641	45%	435	64%	
Family PIR	36,055	2.5 (1.6)	2,960	2.2 (1.5)	*F* = 120.165, *p* < 0.001	7,500	2.5 (1.6)	637	2.2 (1.5)	*F* = 20.628, *p* < 0.001
Alcohol (gm) *	36,582	10 (29)	3,007	6.7 (22)	*F* = 43.14, *p* < 0.001	7,626	10 (30)	641	7.3 (23)	*F* = 7.04, 0.008
Creatinine (mg/dL)	36,990	0.89 (0.43)	3,033	1.1 (0.63)	*F* = 707.711, *p* < 0.001	7,619	0.89 (0.4)	633	1.1 (0.59)	*F* = 177.766, *p* < 0.001
BUN (mg/dL)	36,986	13 (5.7)	3,033	18 (9)	*F* = 1572.97, *p* < 0.001	7,619	13 (5.5)	633	17 (9.2)	*F* = 376.725, *p* < 0.001

*Average consumed during the past 30 days. IHD, ischemic heart disease; PIR, poverty income ratio - a ratio of family income to poverty threshold; SD, standard deviation; PFAS, perfluoroalkyl and polyfluoroalkyl substances.

Moreover, the gender differences in the risk of PFAS exposure between participants with and without IHD were compared (Table 2), as well as the Kaplan–Meier survival analysis. The results are shown in Figures 2C,D. In IHD patients, male subjects had a higher risk of PFAS exposure than female subjects, HR = 1.2 (95%CI: 1.0–1.5, *p* = 0.042), and in non-IHD participants, male subjects had a higher risk of PFOSA exposure than female subjects, HR = 1.3 (95%CI: 1.2–1.5, *p* < 0.001). The performance AUC values of PFAS in distinguishing the survival status in IHD patients (Figure 2C) and controls (Figure 2D) were 0.821 (95%CI: 0.787–0.855) and 0.819 (0.803–0.855), respectively.

**Table 2:** Basic information of participants exposed to PFAS.

Category	Without IHD		With IHD		Test	Female IHD		Male IHD		Test
	*N*	%/Mean (SD)	*N*	%/Mean (SD)		*N*	%/Mean (SD)	*N*	%/Mean (SD)	
Age	8,167	48 (18)	684	68 (13)	*F* = 793.554, *p* < 0.001	261	67 (14)	423	69 (12)	*F* = 1.395, *p* = 0.238
Gender	8,167		684		*X*^2^ = 53.402, *p* < 0.001	261		423		Not available
Female	4,310	53%	261	38%		261	100%	0	0%	
Male	3,857	47%	423	62%		0	0%	423	100%	
Race	8,167		684		*X*^2^ = 65.72, *p* < 0.001	261		423		*X*^2^ = 3.657, *p* = 0.454
Mexican American	1,398	17%	76	11%		30	11%	46	11%	
Non-Hispanic Black	1820	22%	105	15%		42	16%	63	15%	
Non-Hispanic White	3,736	46%	422	62%		152	58%	270	64%	
Other Hispanic	628	8%	42	6%		21	8%	21	5%	
Other Race	585	7%	39	6%		16	6%	23	5%	
Citizenship	8,146		684		*X*^2^ = 55.568, *p* < 0.001	261		423		*X*^2^ = 2.556, *p* = 0.11
Not US	1,235	15%	32	5%		17	7%	15	4%	
US	6,911	85%	652	95%		244	93%	408	96%	
Marital	8,159		684		*X*^2^ = 145.354, *p* < 0.001	261		423		*X*^2^ = 58.104, *p* < 0.001
Married or cohabiting	4,862	60%	385	56%		103	39%	282	67%	
Never married	1,550	19%	37	5%		11	4%	26	6%	
Widowed, divorced or separated	1747	21%	262	38%		147	56%	115	27%	
Pregnancy	2,207		35		*X*^2^ = 1.183, *p* = 0.277	35		0		Not available
No	1991	90%	34	97%		34	97%	0		
Yes	216	10%	1	3%		1	3%	0		
Education	8,149		684		*X*^2^ = 69.346, *p* < 0.001	261		423		*X*^2^ = 9.172, *p* = 0.002
High school and below	4,088	50%	457	67%		193	74%	264	62%	
More than high school	4,061	50%	227	33%		68	26%	159	38%	
FamilyPIR	7,500	2.5 (1.60)	637	2.20 (1.50)	*F* = 20.628, *p* < 0.001	238	2 (1.40)	399	2.4 (1.50)	*F* = 13.971, *p* < 0.001
Total persons in family	8,167		684		*X*^2^ = 27.44, *p* < 0.001	261		423		*X*^2^ = 0.001, *p* = 0.973
<=5	7,236	89%	651	95%		249	95%	402	95%	
>5	931	11%	33	5%		12	5%	21	5%	
PFOS	7,467	17 (18)	610	23 (28)	*F* = 57.9, *p* < 0.001	223	18 (20)	387	26 (32)	*F* = 9.685, *p* = 0.002
PFOA	7,467	3.9 (3.30)	610	4.40 (3.30)	*F* = 9.082, *p* = 0.003	223	4.10 (2.80)	387	4.5 (3.50)	*F* = 2.47, *p* = 0.117
PFHS	7,467	2.4 (3.00)	610	2.50 (2.80)	*F* = 0.439, *p* = 0.507	223	2.10 (1.90)	387	2.7 (3.20)	*F* = 6.252, *p* = 0.013
EPAH	7,467	0.15 (0.14)	610	0.18 (0.20)	*F* = 18.935, *p* < 0.001	223	0.18 (0.13)	387	0.18 (0.23)	*F* = 0.004, *p* = 0.95
MPAH	7,467	0.41 (0.83)	610	0.56 (0.65)	*F* = 18.113, *p* < 0.001	223	0.47 (0.49)	387	0.61 (0.73)	*F* = 5.915, *p* = 0.015
PFDE	7,467	0.4 (0.65)	610	0.44 (0.52)	*F* = 2.329, *p* = 0.127	223	0.38 (0.40)	387	0.47 (0.58)	*F* = 4.26, *p* = 0.039
PFBS	7,467	0.11 (0.13)	610	0.13 (0.10)	*F* = 6.519, *p* = 0.011	223	0.13 (0.10)	387	0.12 (0.10)	*F* = 1.882, *p* = 0.171
PFHP	7,467	0.21 (0.24)	610	0.21 (0.14)	*F* = 0.027, *p* = 0.87	223	0.21 (0.11)	387	0.21 (0.16)	*F* = 0.041, *p* = 0.839
PFNA	7,467	1.4 (1.60)	610	1.60 (1.40)	*F* = 6.127, *p* = 0.013	223	1.30 (1.10)	387	1.7 (1.60)	*F* = 9.452, *p* = 0.002
PFOSA	7,467	0.093 (0.11)	610	0.11 (0.11)	*F* = 11.927, *p* < 0.001	223	0.10 (0.07)	387	0.12 (0.13)	*F* = 3.049, *p* = 0.081
PFUA	7,467	0.29 (0.57)	610	0.33 (0.54)	*F* = 3.512, *p* = 0.061	223	0.26 (0.40)	387	0.37 (0.60)	*F* = 5.737, *p* = 0.017
PFDO	7,467	0.22 (0.24)	610	0.25 (0.25)	*F* = 13.53, *p* < 0.001	223	0.27 (0.26)	387	0.24 (0.25)	*F* = 2.428, *p* = 0.12

IHD, ischemic heart disease; PIR, poverty income ratio - a ratio of family income to poverty threshold; SD, standard deviation; PFAS, perfluoroalkyl and polyfluoroalkyl substances; PFOSA, perfluorooctane sulfonamide; PFOS, perfluorooctane sulfonic acid; PFOA, perfluorooctanoic acid; MPAH, 2-(N-methyl-PFOSA) acetate; EPAH, 2-(N-ethyl-PFOSA) acetate; PFHS, Perfluorohexane sulfonic acid; PFBS, Perfluorobutane sulfonic acid; PFDO, Perfluorododecanoic acid; PFUA, Perfluoroundecanoic acid; PFDE, Perfluorodecanoic acid; PFHP, Perfluoroheptanoic acid; PFNA, Perfluorononanoic acid.

The sex differences in Pearson correlation of PFAS with serum and its derived biomarkers are shown in Figure 2E. Interestingly, in results for total cholesterol (TC), HDL-cholesterol (HDL-C), LDL-cholesterol (LDL-C), serum creatinine (SCR), and blood urea nitrogen (SBU), we found that the correlations with PFOS and PFOA were significantly higher in female subjects than in male subjects. The concentration of MPAH was found to be positively correlated with HDL-C, SCR and SBU, as well as with derived biomarkers such as the systematic immune-inflammation index (SII) systematic inflammation response index (SIRI), and fibrosis-4 score (FIB-4).

### 3.3 Sex differences in dose–response to PFAS

**Figure 3:**
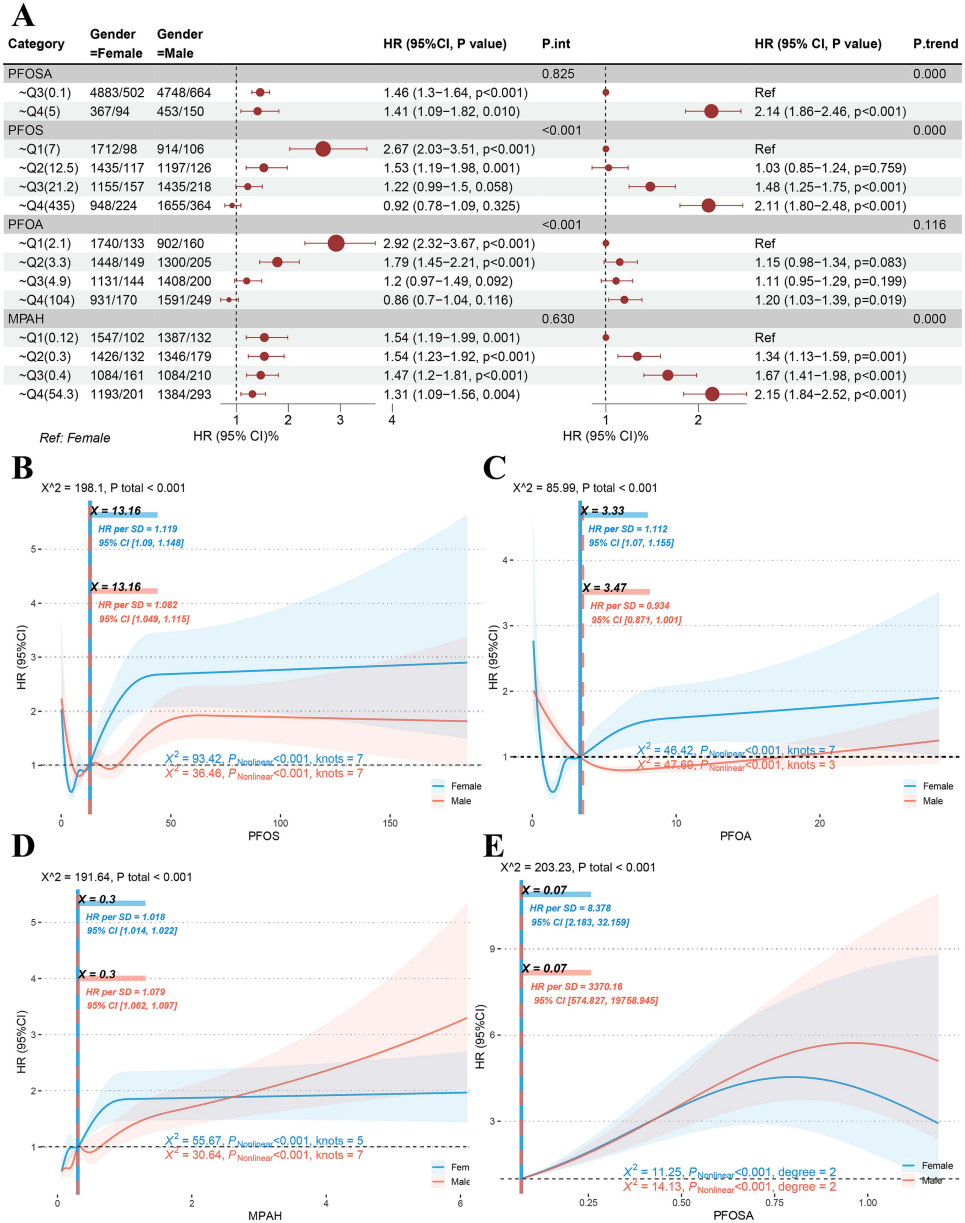
Sex differences in dose–response to PFAS. **(A)** Quantile comparison and regression of male and female populations at risk of exposure to PFAS including PFOSA, PFOS, PFOA and MPAH. **(B-D)** Sex differences in restricted cubic spline models of PFOS, PFOA and MPAH. **(E)** Sex differences in quadratic model of PFOSA. (PFASm per- and poly-fluoroalkyl substances. PFOSA, perfluorooctane sulfonamide; PFOS, perfluorooctane sulfonic acid; PFOA, perfluorooctanoic acid; MPAH, 2-(N-methyl-PFOSA) acetate).

Quantile comparisons of participants at risk of PFAS exposure showed that lower Q1 and Q2 quantiles of PFOS and PFOA worsened survival in male subjects, while higher Q3 and Q4 quantiles had no significant effect (Figure 3A). In contrast, the higher quantiles Q3 and Q4 in PFOSA showed a significantly high hazard ratio. The quantile trend regression results showed that the survival of quantile Q4 was significantly worse than Q1 under PFOSA and PFOS exposures, whereas there was no significant trend in quantiles of PFOA. The results of quantile comparison and trend regression of MPAH showed that the survival rate of male patients decreased significantly.

In RCS models of PFOS and PFOA, significant dose effects were found in both male and female populations. As shown in Figure 3B, the RCS curve for PFOS showed the same S-shapes and the same inflection point of 13.16. However, the high level of HR suggests that women (HR = 1.119, 95%CI: 1.09–1.148) may be more sensitive to the toxic response of this substance than men (HR = 1.082, 95%CI: 1.049–1.115). Interestingly, female subjects showed an L-S-shaped response to PFOA (Figure 3C), with an inflection point of 3.33 (HR = 10,112, 95%CI: 1.07–1.155), while male subjects showed an L-shaped response, with an inflection point of 3.47 (HR = 0.934, 95%CI: 0.871–1.001). In results for MPAH (Figure 3D), female subjects showed an S-shape (HR = 1.018, 95%CI: 1.014–1.022), while male subjects showed a J-shape (HR = 1.079, 95%CI: 1.052–1.097) correspond to the significant reasons for the aforementioned quantile comparison and trend regression. The RCS model fit for PFOSA failed because it had fewer than three nodes. Thus, a quadratic model (Figure 3E) was used to investigate its dose effect on hazard ratio, and significant non-linear associations were found for both sexes.

### 3.4 Results of functional annotation of genes affected by PFAS

**Figure 4:**
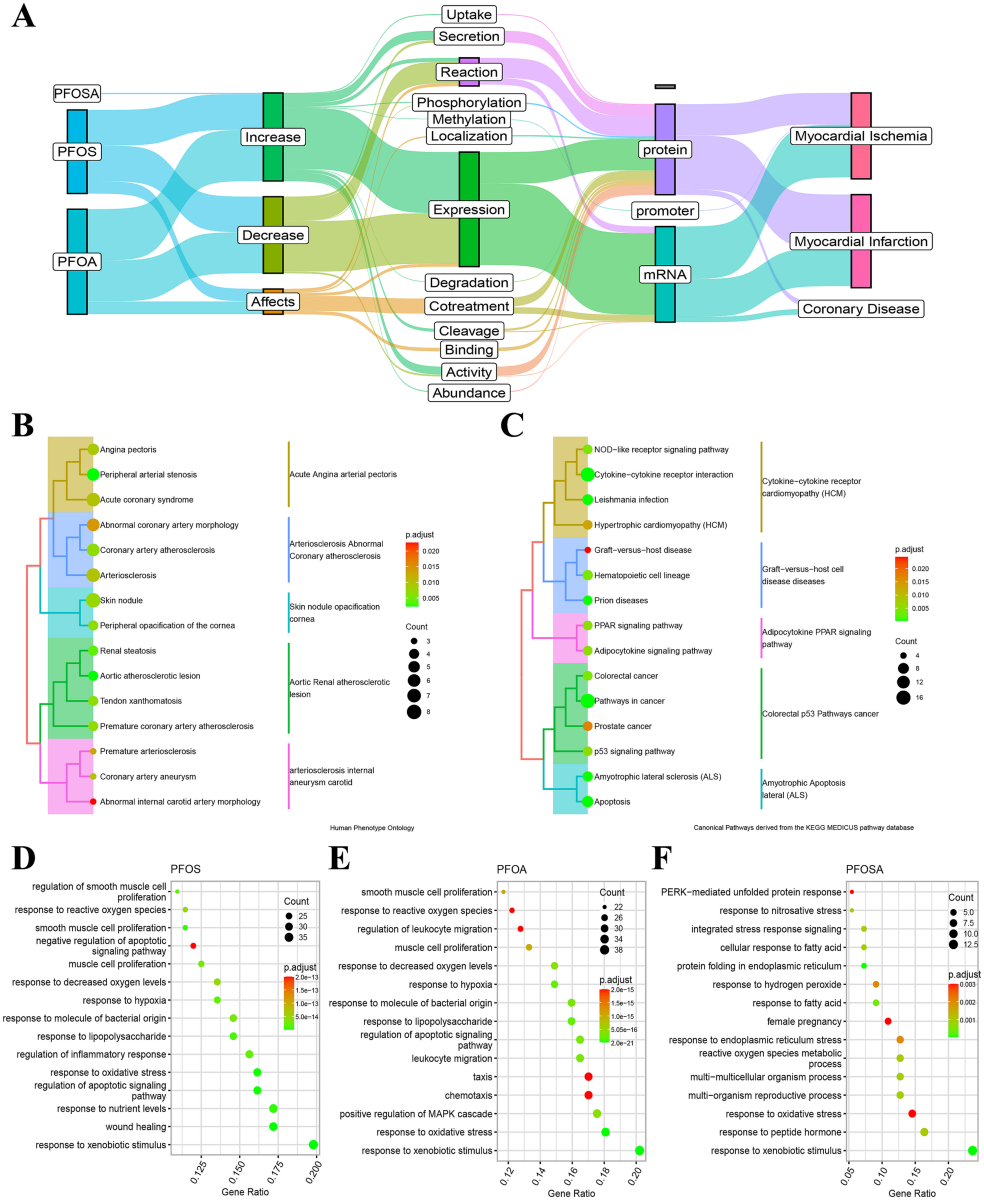
Functional annotation of PFAS affected genes. **(A)** Alluvial maps of the interactions and phenotypes of human genes affected by PFOSA, PFOS and PFOA. **(B)** Human phenotype ontology annotation of PFAS affected genes. **(C)** Kyoto Encyclopedia of Genes and Genomes annotation of PFAS affected genes. **(D-F)** Biological processes ontology annotation of affected genes by PFOS, PFOA and PFOSA. The dot size and color bar represent the number of mapped genes and the adjust *p*-value, respectively. (PFAS, per- and poly-fluoroalkyl substances; PFOSA, perfluorooctane sulfonamide; PFOS, perfluorooctane sulfonic acid; PFOA, perfluorooctanoic acid; MPAH, 2-(N-methyl-PFOSA) acetate).

According to CTDbase, a total of 105 human genes are affected by PFAS (including PFOSA, PFOA, and PFOS, except MPAH) (Supplementary Table S2). An overview of the interactions and phenotypes affected by PFOSA, PFOS, and PFOA was shown in Figure 4A. In myocardial infarction or ischemic disease, PFOA and PFOS increase or decrease the expression of most genes at the protein and mRNA levels.

Consistent with the evidence obtained from CTDbase, the HPO annotation of target genes mainly focused on cardiovascular diseases (Figure 4B), such as angina pectoris, acute coronary syndrome, aortic atherosclerotic lesion, coronary artery atherosclerosis, etc. In the first 15 annotations of KEGG (Figure 4C), the function of these genes primarily affects signaling pathways, including NOD-like receptor, adipocytokine, peroxisome proliferator-activated receptor (PPAR), and p53, as well as diseases, including prion diseases, graft-versus-host disease, amyotrophic lateral sclerosis, colorectal cancer, prostate cancer, and hypertrophic cardiomyopathy.

In addition, we annotated the biological processes of PFOS-, PFOA- and PFOSA-affected genes, separately (Figures 4D–F). For the first 15 annotated results of each enrichment, the effects of the three chemicals showed some similarity in biological function, such as response to reactive oxygen species, oxidative stress, xenobiotic stimulus, hypoxia, and fatty acid, as well as the regulation of leukocyte migration and smooth muscle cell proliferation.

### 3.5 Network interactions and molecular docking scores of PFAS affected genes

**Figure 5:**
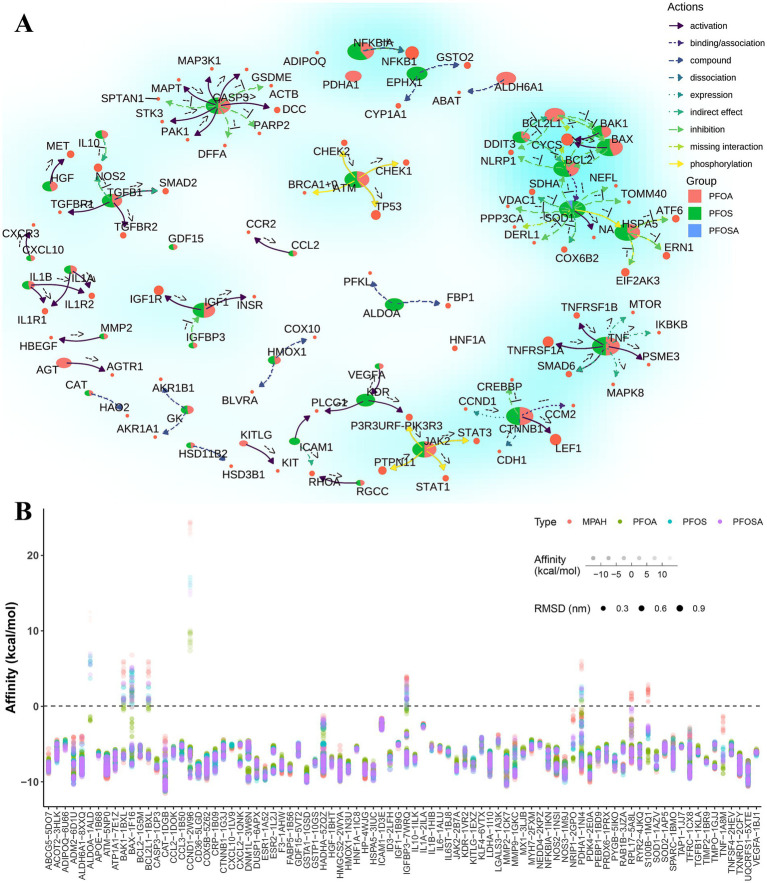
The network interactions and molecular docking scores of PFAS affected genes. **(A)** Network interactions of PFAS affected genes and target genes based on KEGG pathways. Pie chart represent the proportion of the number of adjacent edges (degree > = 2) of each PFAS. The colored arrows indicate the type of gene action as well as the direction of action. The exact type of gene direct regulation is indicated by the annotations near the arrows. For example, the indicators “-->,” “--|” and “− + −” represent “activation,” “inhibition” and “dissociation,” respectively. **(B)** Results of molecular docking of optimal protein-binding pockets of affected genes with PFAS. Dot size, color and transparency represent the RMSD, PFAS and affinity, respectively. (PFAS, per- and poly-fluoroalkyl substances; PFOSA, perfluorooctane sulfonamide; PFOS, perfluorooctane sulfonic acid; PFOA, perfluorooctanoic acid; MPAH, 2-(N-methyl-PFOSA) acetate; RMSD, root mean square deviation).

Based on the KEGG pathways database, we constructed a regulatory network (Figure 5A) of these 105 human genes with downstream target genes, which provides new insights into the study of the mechanism of PFAS exposure affecting IHD. For example, the CASP3 gene, alias for caspase 3, activates the expression of genes such as DCC, PAK1, STK3, and MAPT, while directly suppressing the expression of genes such as DFFA, PARP2, GSDME, and SPTAN1.

To further understand the potential effects of PFAS on these human genes, molecular docking methods were widely applied to available binding pockets of all crystal structures with drug scores greater than 0.1. As shown in Figure 5B, most of the docking complexes obtained high affinity scores, with an average value of −7.3 kcal/mol and an average root mean square deviation (RMSD) of 0.29 nm.

### 3.6 Evaluation of significant genes docking complexes with PFAS

**Figure 6:**
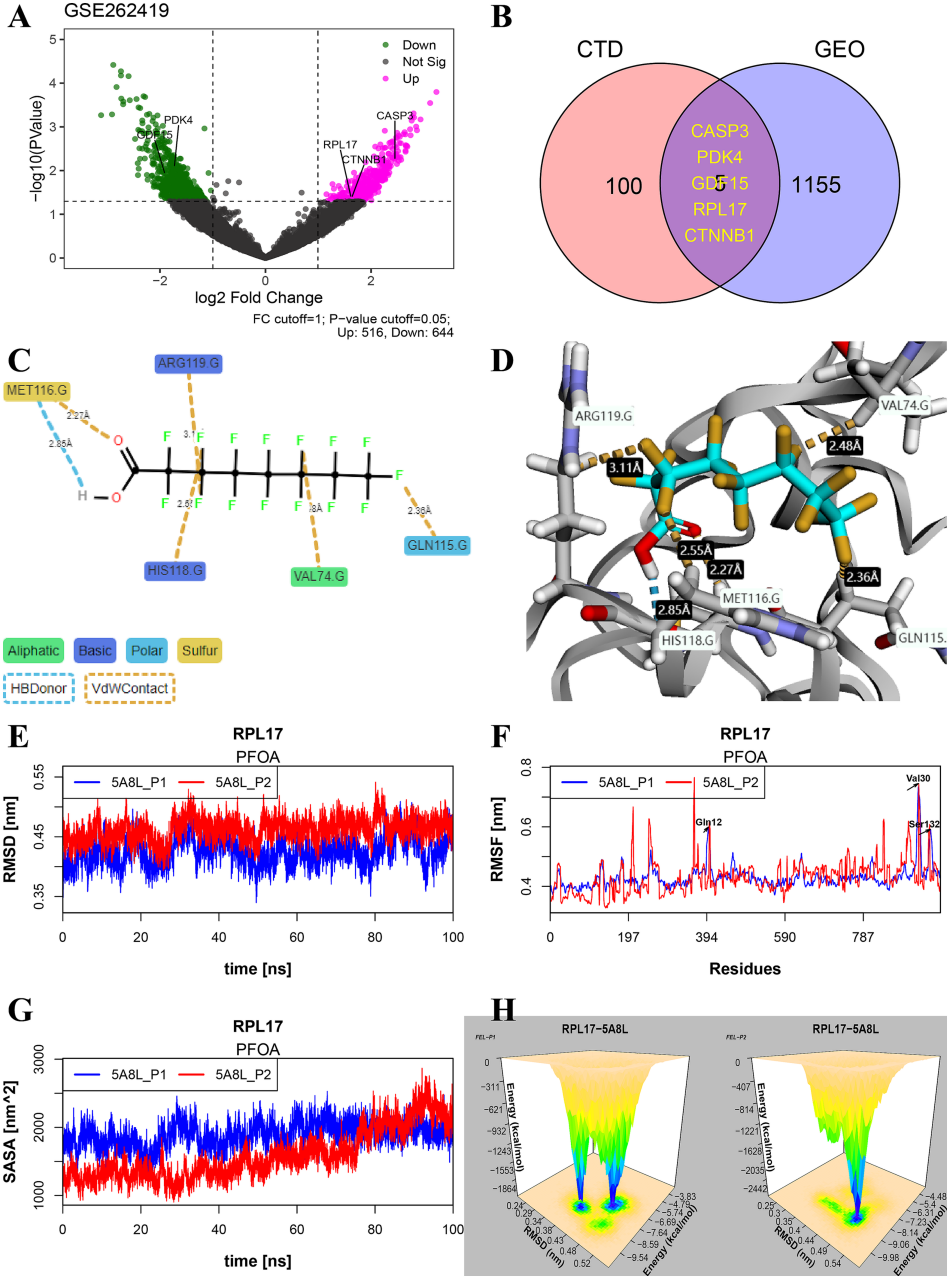
Identification of significant genes and study on their binding stability. **(A)** Volcano plot of differentially expressed genes (DEGs) in dataset GSE262419. **(B)** The significant common genes of PFAS affected genes in CTDbase and DEGs in GSE262419. **(C,D)** Example maps of 2D and 3D receptor-ligand interactions for PFOA and RPL17 (PDB: 5ABL) crystal structure. **(E–H)** Molecular dynamics simulation results of the two optimal docking postures for molecules 5ABL-PFOA, including RMSD **(E)**, RMSF **(F)**, SASA **(G)**, and 3d-FEL **(H)**. The 3d-FEL was generated based on the RMSD and FEL during 100 ns dynamics simulations, and the peaks showed the stable binding mode of the complex. (PFAS, per- and poly-fluoroalkyl substances; PFOA, perfluorooctanoic acid; RMSD, root mean square deviation; RMSF, root mean square fluctuation; SASA, solvent accessible surface area; FEL, free energy landscape).

In PFAS-treated PSCC-derived datasets, 516 upregulated and 644 downregulated DEGs were found based on log2FoldChange greater than 1 and *p* values less than 0.05 (Figure 6A). Combined with PFAS-affected genes in CTDbase (Figure 6B), five significant genes were identified, including CASP3, PDK4, GDF15, RPL17, and CTNNB1.

In this study, the PFOA-5ABL docking complex was used as an example to evaluate the potential long-term toxicity of PFAS. As shown in Figure 6C, hydrogen bonds and sulfur contacts were formed between the carboxyl group and MET116 at distances of 2.85 Å and 2.27 Å, respectively. The stereo-binding conformation of the complex is shown in Figure 6D. The RMSD varied from 0.36 to 0.53 nm, indicating the complex maintained high stability during 100 ns of simulation (Figure 6E). The position changes of all amino acids are represented by root mean square fluctuation (RMSF) (Figure 6F), which reflects the flexibility of the complex’s conformational movement during the simulation. Additionally, solvent accessible surface area (SASA) is another tool used to assess the flexibility of the conformation of complexes in contact with a solvent and to predict the magnitude of binding-induced conformational changes (Figure 6G). In the simulation results of 100 ns of PFOA-5ABL, the free energy landscape (FEL) was meshed with 50 bins combined with RMSD, and the relationship between the two in the simulated environment was visualized by stereogram (Figure 6H). The peak location represents the relatively stable FEL and RMSD values of the complex.

## 4 Discussion

In the present study, we summarized the sex differences in IHD mortality, morbidity, YLDs, and YLLs in different SDI regions worldwide. Although the health problems caused by IHD are decreasing globally, they remain significant, especially in low-SDI regions. Consistent with the previous ISCHEMIA randomized clinical trial (26), we observed less ischemia in female participants based on the NHANES dataset. What’s more, the same gender disparities in PFAS exposures were found in both IHD and the control subjects. Subsequently, several PFAS substances, including PFOSA, PFOS, PFOA, and MPAH, were identified as important contributors to the risk of IHD. Finally, quantile regression and RCS models verified the sex differences in the risk of dose-effect exposure to PFAS.

PFAS are widely used in consumer and industrial products because of their unique structure and excellent properties, but their toxic reactions also cause long-term harm to biological systems (27, 28). PFAS in the environment are resistant to degradation under natural conditions and microbial activity (29), thus migrating in the environment and accumulating in biota (30), and particularly volatile PFAS facilitate long-distance transport. In recent perspectives, legacy PFAS (e.g., PFOA and PFOS) exposure constitutes cardiovascular toxicity to human health (6, 7, 31), as well as hypertension (3, 11, 32), endocrine dysfunction (2, 33), lipid metabolism (34–36), and cancer (1, 37). For example, a nationally representative cross-sectional study conducted in China (13), including 10,855 participants over the age of 18, focused on the effects of PFAS on lipid metabolism. The results showed a positive correlation with TC, HDL, and LDL. Our findings not only confirmed these associations but also revealed the existence of sex differences.

Currently, the mechanism of PFAS on IHD is controversial. The dyslipidemia caused by legacy PFAS, particularly PFOA and PFOS, has been demonstrated in several animal experiments (38, 39). The primary mechanisms probably involve the absorption and catabolism of fatty acids, as well as catalase and glutathione S-transferase activities (40, 41). In a previous *in vivo* study, which included 290 individuals exposed to PFOA and PFOS through drinking water, gene expression of NR1H2, NPC1, and ABCG1 was found to be negatively correlated with the concentration of PFOS, but positively correlated with NCEH1 and PPAR*α* in women alone (41). The functions of these affected genes are involved in adipocyte differentiation, plasma lipoprotein assembly, remodeling, and clearance (42–44). The accumulation of TC, LDL-C, and HDL-C induced by exposure to PFAS constitutes a hypercholesterolaemic environment, which is a major contributor to arteriosclerosis. More importantly, the toxicity of PFAS to pregnant women, infants, and children is a warning that the whole society needs to pay attention to environmental pollutants (31, 34–36, 45).

The main highlight of this study is the identification of new PFAS-affected genes through multiple databases, which not only reveals a series of significant mechanisms involved in the biological processes of IHD but also evaluates their potential binding postures and affinities through molecular docking. For example, the cysteine protease CASP3 gene (another name for caspase 3) is highly expressed in PSCC cell lines treated with PFAS and is an important target for PFAS (mean affinity −8.63 kcal/mol). These suggest that PFAS exposure plays an important role in activating apoptotic signaling transduction and becomes a risk factor for the pathogenesis of IHD. Most recently, the CASP3 gene was shown to be overexpressed in the brains of carp exposed to PFAS, while causing overexpression of the pro-inflammatory cytokine TNF-α, IFN-*γ*, and the stress-related gene HSP-70 (46). In a recently study, in the testicles and epididymis of rats exposed to PFOA, oxidative damage is triggered and CASP3 mRNA is upregulated, which may lead to male infertility (47). Furthermore, the PFOA toxicity affects the development and growth of ovarian follicles through CASP3 (48), as well as ameloblasts and tooth enamel formation (49).

PFOS and PFOA induce the expression of pyruvate dehydrogenase kinase 4 (PDK4), which plays a key role in regulating glucose and fatty acid metabolism, leading to an increase in fatty acid oxidation products (50). *In vitro* study by Zhange et al., showed that inhibition of PDK4 or knockdown of PDK4 can effectively attenuate the mitochondrial toxicity of PFOA in human liver and enterocytes (51). Thus constitutes an oxidative stress and inflammatory environment, which are known to be the two major contributors to arteriosclerosis (52–54). They also found that compared with PFOA exposure, the modification of carboxyl groups by PFOA esterification of methyl perfluorooctanoate led to the loss of upregulated expression of PPARα and PDK4, and reduced mitochondrial toxicity and cytotoxicity (51).

GDF15 (an alias for growth differentiation factor 15) was proven to be positively associated with mixed exposure to PFAS in a study that included 312 overweight or obese adolescents from the Study of Latino Adolescents at Risk (55). Two other important genes, CTNNB1 and RPL17, play important roles in constituting adhesive junctions and catalyzing protein synthesis, respectively, but have not been identified as PFAS target genes.

The long-term toxicity of PFAS is not only caused by its unique properties but also by its inefficient elimination in animals and humans, leading to its continuous accumulation. PFAS elimination is largely dependent on non-metabolic pathways, such as enterohepatic circulation of bile acids, urine, and feces, which are thought to contribute to the elimination of PFAS in human serum (8–10). However, organic anion transporter 4 and urate transporter 1 are considered key transporters for renal reabsorption of perfluorocarboxylates, which is one of the reasons for the long half-life of PFAS in humans (56). Yet, more than any of these, the food chain and the water cycle may contribute significantly to the accumulation of PFAS in humans, at least as re-exposure to metabolites from water has been shown to be a major uptake route for fish (57).

Since there is no effective way to remove legacy PFAS, prevention and treatment may be important approaches to address PFAS-related diseases in the future. The silencing of PPARα in PFOA-induced chicken embryo heart models suggests that it has a protective effect on cardiomyocyte viability and cell morphology (58, 59). Meanwhile, L-carnitine has antioxidant and NO regulatory effects, which may protect cardiomyocytes from the toxic effects of PFOA (58, 60). Our study suggests that the genes GK (glycerol kinase) and ADIPOQ (adiponectin) in the PPAR signaling pathway (Figure 4C) may be targets of PFAS, and the molecular docking results showed good binding scores. In addition, proteins encoded by GK play an important role in triglyceride metabolism, and proteins encoded by ADIPOQ circulate in plasma and are involved in metabolic processes, such as responses to oxidative stress and hypoxia (Figures 4D–F).

This study has the following limitations in terms of methods and results: (a) Results were not verified in other large cohort datasets. (b) Paradoxically, female IHD patients are more susceptible to PFAS toxicity than male patients, while male patients have significantly lower survival rates than female patients. This may be due to confounding factors and sex differences inherent in complex diseases (26, 61). (c) Participants’ self-reported disease status is inadequate compared with professional diagnosis. (d) The causal relationship between PFAS and IHD could not be confirmed based on these analysis results. (e) Molecular docking and dynamics simulations are evaluated with only one software and algorithm, which may weaken the power of the conclusion.

## 5 Conclusion

Our study reinforces the gender differences in IHD patients in different SDI regions around the world, as well as in toxic responses to PFAS exposures, and provides a series of PFAS receptor genes associated with cardiovascular disease that may influence key pathways in IHD pathogenesis. The susceptibility of women to the toxicity of PFOA and PFOS is a warning that we should pay more attention to women’s healthy. Finally, five important genes, including CASP3, PDK4, GDF15, RPL17, and CTNNB1, are considered potential targets for the prevention and treatment of PFAS-associated IHD in future studies.

## Data Availability

Publicly available datasets were analyzed in this study. This data can be found here: all data is available through the Global Health Data Exchange (GHDx), National Health and Nutrition Examination Survey (NHANES), Comparative Toxicogenomics Database (CTDbase) and Gene Expression Omnibus (GEO) database.

## Footnotes

^1^ https://www.rcsb.org/

^2^ https://cloud.r-project.org/

## Glossary

IHD: ischemic heart disease
RCS: restricted cubic spline.
PFAS: per- and poly-fluoroalkyl substances
PFOSA: perfluorooctane sulfonamide
PFOS: perfluorooctane sulfonic acid
PFOA: perfluorooctanoic acid
MPAH: 2-(N-methyl-PFOSA) acetate
TC: total cholesterol
HDL-C: HDL-cholesterol
LDL-C: LDL-cholesterol
SAL: serum albumin
SCR: serum creatinine
SBU: serum blood urea nitrogen
AIP: atherosclerosis index of plasma
SII: systematic immune-inflammation index
SIRI: systematic inflammation response index
FIB-4: fibrosis-4 score
CASP3: caspase 3
GDF15: growth differentiation factor 15
PDK4: pyruvate dehydrogenase kinase 4
GHDx: Global Health Data Exchange
NHANES: National Health and Nutrition Examination Survey
CTDbase: Comparative Toxicogenomics Database
GEO: Gene Expression Omnibus
RMSD: root mean square deviation
RMSF: root mean square fluctuation
SASA: solvent accessible surface area
FEL: free energy landscape
PPAR: peroxisome proliferator-activated receptor
