# Supplementary figures and images for: Sex differences in ischemic heart disease and evidence gathering related to exposure risk, prevention, and treatment of per- and poly-fluoroalkyl substances
Source: Front Public Health. 2025 Jun 20;13:1596125. doi: 10.3389/fpubh.2025.1596125 (PMC12226470; doi:10.3389/fpubh.2025.1596125)

## Hazard ratios of candidates

● Multivariate ● Univariate

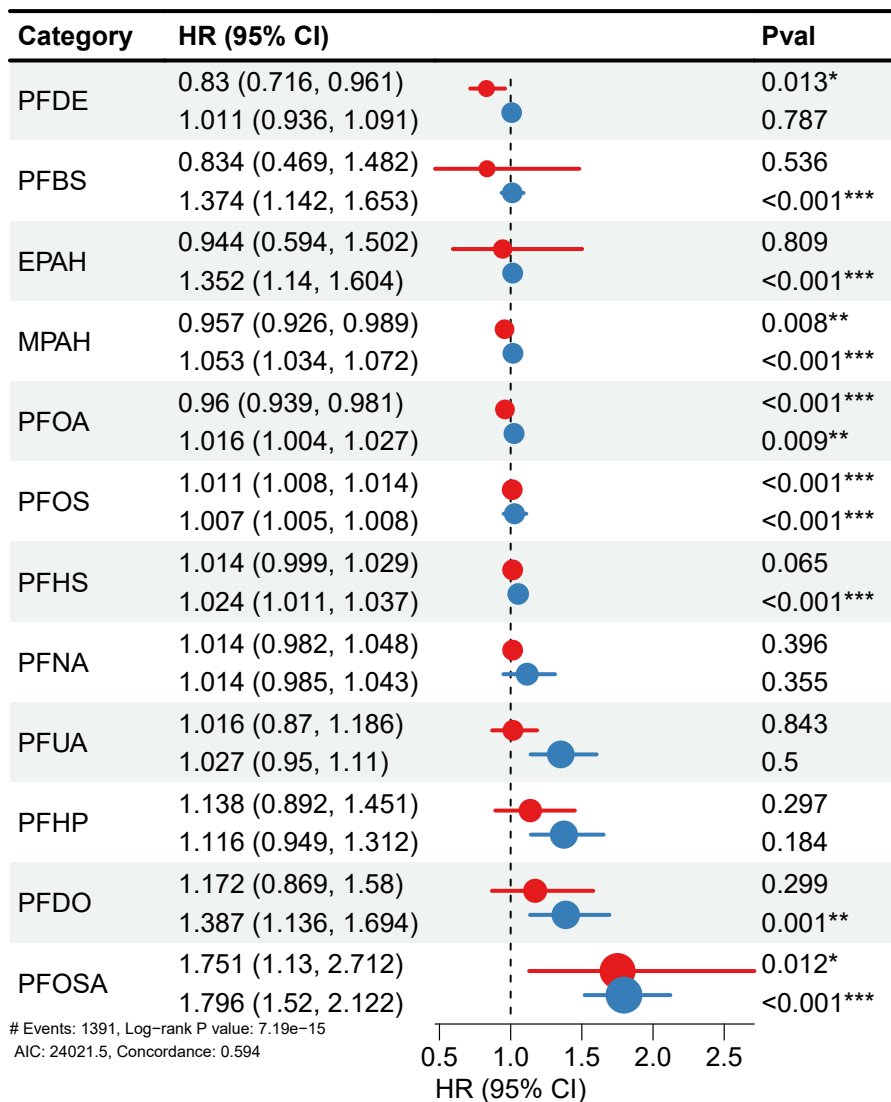

Supplement: Supplementary file 1 [file Data_Sheet_1.pdf]
